# GDP (Gemcitabine, Dexamethasone, and Cisplatin) Is Highly Effective and Well-Tolerated for Newly Diagnosed Stage IV and Relapsed/Refractory Extranodal Natural Killer/T-Cell Lymphoma, Nasal Type

**DOI:** 10.1097/MD.0000000000002787

**Published:** 2016-02-12

**Authors:** Jing-jing Wang, Mei Dong, Xiao-hui He, Ye-xiong Li, Wei-hu Wang, Peng Liu, Jian-liang Yang, Lin Gui, Chang-gong Zhang, Sheng Yang, Sheng-yu Zhou, Yuan-kai Shi

**Affiliations:** From the Department of Medical Oncology (J-JW, MD, X-HH, PL, J-LY, LG, C-GZ, SY, S-YZ, Y-kS); and Department of Radiation Oncology, Cancer Hospital, Chinese Academy of Medical Sciences (CAMS) and Peking Union Medical College (PUMC), Chaoyang, Beijing, China (Y-XL, W-HW).

## Abstract

This study was conducted to evaluate the effectiveness and tolerance of GDP (gemcitabine, dexamethasone, and cisplatin) regimen in patients with newly diagnosed stage IV and relapsed/refractory extranodal natural killer/T-cell lymphoma, nasal type (ENKTL).

The study enrolled 41 ENKTL patients who received GDP regimen at the Cancer Hospital, Chinese Academy of Medical Sciences and Peking Union Medical College between January 2008 and January 2015.

The disease status was newly diagnosed stage IV in 15 patients and relapsed/refractory in 26 patients. The median number of cycles of chemotherapy per patient was 6 (range, 2–8 cycles). The overall response rate and complete-remission rate were 83.0% (34/41) and 41.5% (17/41), respectively. After a median follow-up of 16.2 months, 1-year progression-free survival rate and 1-year overall survival rate for the whole cohort were 54.5% and 72.7%. Grade 3 to 4 adverse events included neutropenia (34.1%), thrombocytopenia (19.5%), and anemia (14.6%).

Our study has suggested high efficacy and low toxicity profile of GDP regimen in patients with newly diagnosed stage IV and relapsed/refractory ENKTL.

## INTRODUCTION

Extranodal natural killer/T-cell lymphoma, nasal type (ENKTL) is a highly aggressive non-Hodgkin lymphoma (NHL). ENKTL shows a geographical predilection for Asian and South American populations and is rare in Europe and North America. It accounts for 12%–17% of NHL and 47%–56% of peripheral T-cell lymphomas (PTCLs) in China.^1,2^ This disease is typically diagnosed at early stage and is primarily located in the upper aerodigestive tract. Concurrent chemoradiotherapy or sandwiched radiotherapy has been recognized as effective treatment for localized ENKTL patients.^3–7^

However, patients with newly diagnosed stage IV and relapsed/refractory disease experience poor long-term survival; these tumors express high levels of p-glycoprotein, rendering them insensitive to anthracycline-containing (CHOP [cyclophosphamide, doxorubicin, vincristine, prednisolone] or CHOP-like regimens) chemotherapy.^8^ Therefore, effective chemotherapeutic regimens are needed to improve the survival outcome of these patients.

Gemcitabine is a pyrimidine antimetabolite and has a chemical structure that is similar to cytarabine. There have been a few studies reporting the effectiveness of gemcitabine in PTCLs.^9–11^ Our previous study reviewed the therapeutic results of gemcitabine-based regimens in patients with PTCLs (including ENKTL) and demonstrated its high activity and low toxicity.^12^ The study has been cited by National Comprehensive Cancer Network guidelines in the treatment of PTCLs. In this study, we aim to assess the efficacy and safety of GDP (gemcitabine, dexamethasone, and cisplatin) chemotherapy regimen in patients with newly diagnosed stage IV and relapsed/refractory ENKTL.

## MATERIALS AND METHODS

### Patients

From January 2008 to January 2015, patients diagnosed with ENKTL who received GDP regimen at the Cancer Hospital, Chinese Academy of Medical Sciences and Peking Union Medical College were systemically reviewed. The inclusion criteria for the study were as follows:

1. Diagnosis of ENKTL with typical morphology and immunophenotype according to the 2008 World Health Organization classification of lymphomas, indicating that the neoplastic cells were negative for B-cell antigens, such as CD20 and CD79α, but they were positive either for CD3ε and CD56, or for CD3ε, cytotoxic molecules (granzyme-B, T-cell intracellular antigen-1, and perforin) and Epstein–Barr virus if cells were CD56-negative
2. Age ≥18 years
3. Eastern Cooperative Oncology Group performance status 0–2
4. At least 1 measurable lesion, defined as a tumor diameter ≥1 cm in computed tomography or MRI scans or ≥2 cm in ultrasound or ≥2 cm by physical examination, before receiving GDP regimen
5. Adequate hematologic, hepatic, and renal functions: absolute neutrophil count ≥1.5 × 10^9^ /L, platelet count ≥100 × 10^9^ /L, total bilirubin ≤1.5 × upper limit of normal, AST and ALT ≤2 × upper limit of normal, and creatinine ≤1.5 mg/dL
6. Newly diagnosed stage IV and relapsed/refractory disease
7. Receiving at least 2 cycles of GDP regimen.

Patients, who did not have complete clinical information or immunohistochemistry or who were lost to follow-up immediately after treatment, were excluded from this study. This study was a retrospective observational study and patients’ information were collected in the hospital database. There was no direct intervention in patients’ treatment or care. Therefore, ethical approval and a patient's consent are not required.

### Disease Evaluation

Clinical evaluations at the time of this study entry included medical history and physical examination, complete blood cell count, serum biochemistry (including hepatic function, renal function, electrolytes, and lactate dehydrogenase), electrocardiogram, and bone marrow examination. Nasopharyngolarygnoscope, magnetic resonance imaging of the head and neck, computed tomography scans of the chest, abdomen, and pelvis areas were implemented. Positron emission tomography was recommended but not compulsory. The clinical features evaluated for potential prognostic importance included disease subtype, stage, disease status, and absolute lymphocyte count (ALC) before administration of GDP regimen. All patients were staged and scored according to the Ann Arbor staging system, the international prognostic index,^13^ and the Korean prognostic index.^14^

### Treatment

GDP regimen was implemented in 21-day cycle: gemcitabine (1000 mg/m^2^ intravenously over 30 minutes on days 1 and 8), dexamethasone (20 mg/day orally on days 1–4 and days 11–14), and cisplatin (25 mg/m^2^ intravenously over 60 minutes on days 1–3). Treatment responses were evaluated after every 2 cycles and at the end of treatment using the international criteria for lymphoma.^15,16^ Complete-remission (CR) was defined as disappearance of all previously measurable lesions and absence of any new tumor lesions. Partial-remission (PR) was defined as a decrease of at least 50% in the product of 2 perpendicular diameters of each measurable lesion. Progressive disease (PD) was defined as greater than 25% increase in the product of the 2 diameters of at least 1 tumor or as the presence of a newly developed lesion. Stable disease (SD) was defined as any response that did not fall into the other defined categories. After chemotherapy, patients could undergo additional palliative radiotherapy. The decision to enroll patients into radiotherapy was based on the physician's discretion, influenced by the patient's disease and performance status, and the patient's own willingness.

### Toxicity Assessments and Dose Modifications

Patients underwent clinical examination, routine complete blood counts and biochemical tests before each new treatment cycle for toxicity evaluation. Toxicities were graded according to the Common Terminology Criteria for Adverse Events version 3.0. If patients developed any grade 3 nonhematological toxicities (except alopecia), grade 4 hematological toxicities, grade 3 neutropenia complicated with fever higher than 38.5 degrees, or grade 3 thrombocytopenia complicated with hemorrhage, doses of gemcitabine and cisplatin were reduced by 25% in successive cycles. If the aforementioned toxicity occurred again, doses were reduced by 50%. Chemotherapy was discontinued with the occurrence of any grade 4 nonhematological toxicities. Granulocyte colony stimulation factor was administered in cases where grade 4 neutropenia or leukopenia and grade 3 neutropenia or leukopenia complicated with fever were observed; interleukin-11 was implemented in cases where grade 4 thrombocytopenia and grade 3 thrombocytopenia complicated with hemorrhage.

### Statistical Analysis

Overall survival (OS) was defined as the period from the 1st day of GDP administration to the date of patient death for any cause or last follow-up. Progression-free survival (PFS) was defined as the period from the 1st day of GDP administration to the date of disease progression or relapse. Both OS and PFS were analyzed using the Kaplan–Meier method. Comparisons of OS or PFS between groups were performed using log-rank test. A 2-tailed *P* < 0.05 was considered statistically significant. Pearson χ^2^ or Fisher exact tests were used to compare the overall response rates (ORR) of different groups. ORR is defined as CR plus PR.

## RESULTS

### Patient Characteristics

Forty-one patients were reviewed in this study. Patient characteristics were summarized in Table 1. The ratio of men to women was 29:12. The median age was 36 years old, and majority of patients presented with Eastern Cooperative Oncology Group performance status at 0–1. Fifteen patients had newly diagnosed stage IV disease, and 26 were relapsed/refractory. For relapsed/refractory patients, previous treatments included radiotherapy alone (N = 8), chemotherapy alone (N = 3), and radiotherapy plus chemotherapy (N = 15). Chemotherapy regimens that were used in previous treatment included CHOP or CHOP-like, modified SMILE (steroid, methotrexate, ifosfamide, pegaspargase, and etoposide), etoposide, ifosfamide, cisplatin, and dexamethasone, and AspaMetDex (pegaspargase, methotrexate, and dexamethasone).

**TABLE 1:**
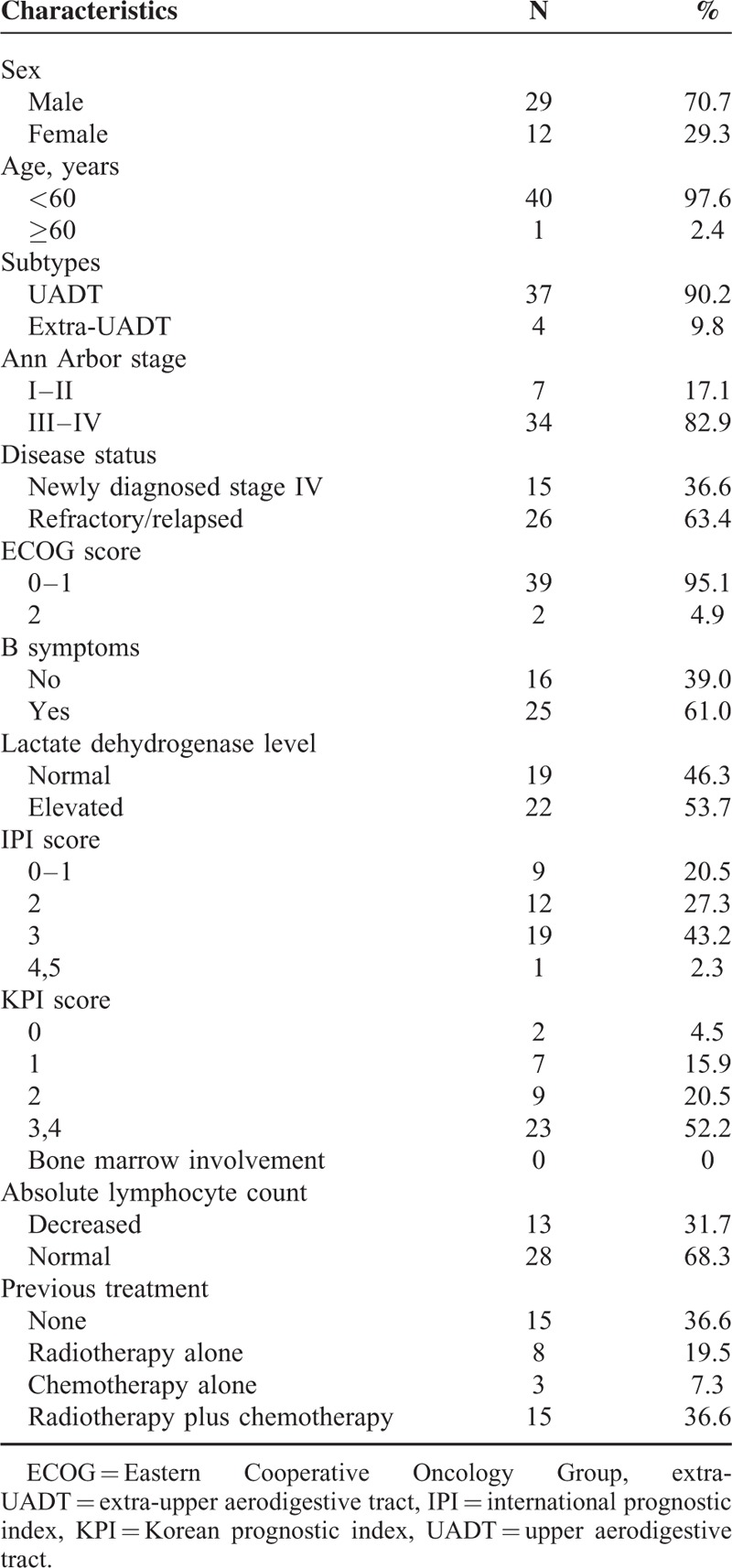
Patient Characteristics

### Response Rates

A total of 196 cycles were administered and the median number of cycles per patient was 6 (range, 2–8 cycles). For 3 patients who received chemotherapy alone in previous treatment and 2 patients who underwent outfield failure, palliative radiotherapy was implemented after GDP chemotherapy.

All patients were evaluable for response to GDP chemotherapy. The rates of CR, PR, SD, and PD were 41.5% (17/41), 41.5% (17/41), 9.8% (4/41), and 7.3% (3/41), respectively. Newly diagnosed patients achieved a higher ORR than relapsed/refractory ones (100.0% vs 73.1%, *P* = 0.035). Patients with normal ALC achieved a higher ORR than patients with decreased ALC (92.9% vs 61.5%, *P* = 0.042) (Table 2). Patients who underwent dose reduction during GDP chemotherapy because of toxicity achieved similar ORR compared with patients who completed GDP chemotherapy without dose reduction (66.7% vs 85.7%, *P* = 0.576).

**TABLE 2:**
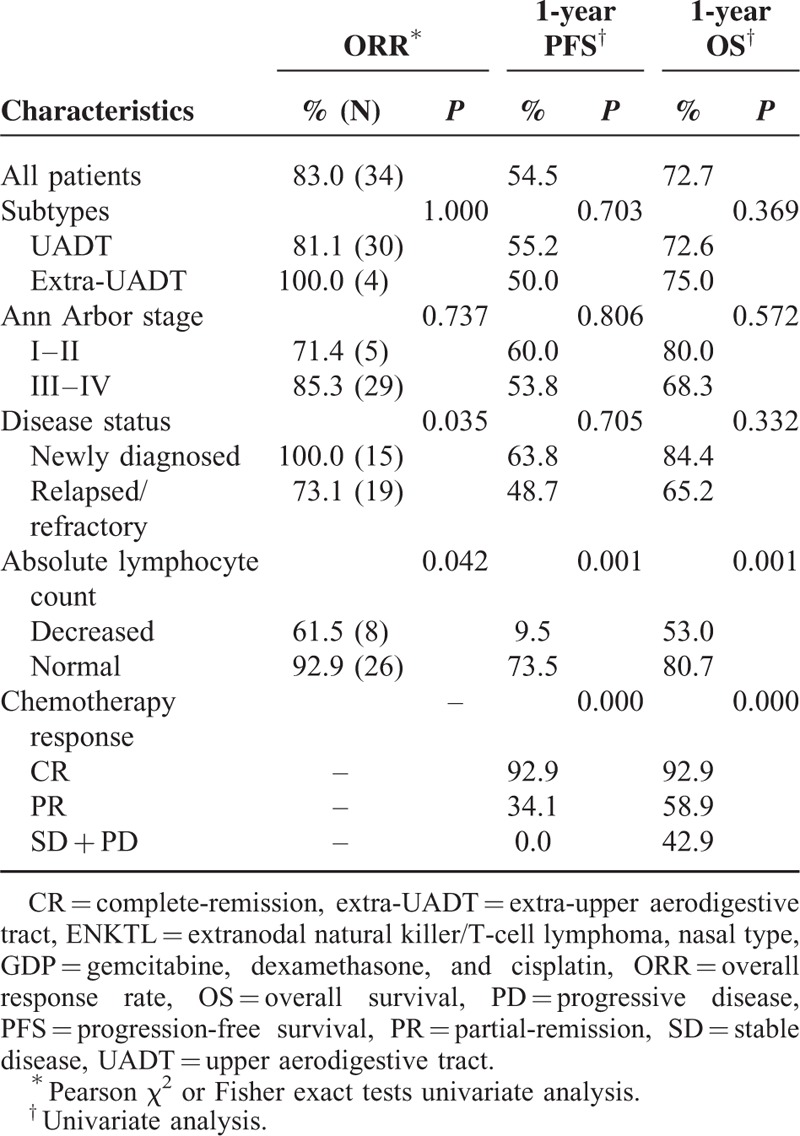
Results of ORR, PFS, and OS With GDP Regimen in ENKTL

### Survival Rates and Prognostic Factors

**FIGURE 1:**
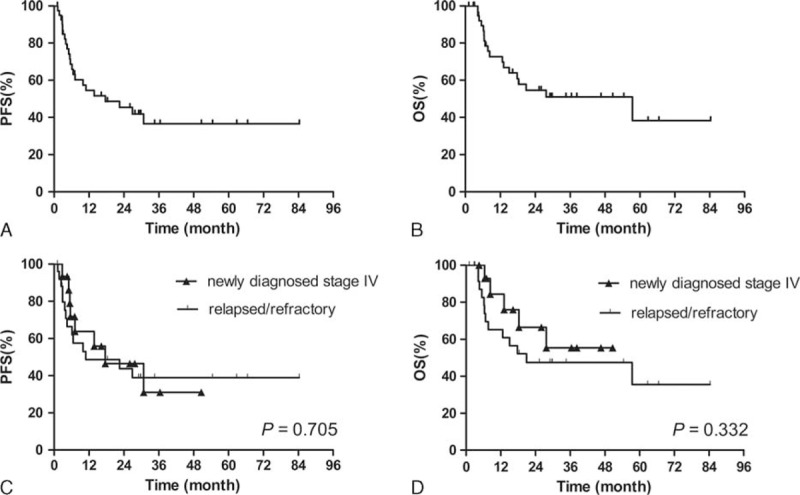
PFS (A) and OS (B) for the whole cohort; comparison of PFS (C) and OS (D) between newly diagnosed stage IV patients and relapsed/refractory patients. OS = overall survival, PFS = progression-free survival.

At a median follow-up of 16.2 months (range, 1.8–96.3 months), 18 patients died of lymphoma, and 8 patients were still alive with disease and 13 patients survived without disease. Two cases were lost to follow-up. The 1- and 2-year PFS rates for all patients were 54.5% and 45.4% (Figure 1A); the 1- and 2-year OS rates for all patients were 72.7% and 54.7% (Figure 1B). No significant differences of PFS and OS were observed between newly diagnosed stage IV patients and relapsed/refractory ones (1-year PFS: 63.8% vs 48.7%, *P* = 0.705, and 1-year OS, 84.4% vs 65.2%, *P* = 0.332) (Figure 1C and D).

**FIGURE 2:**
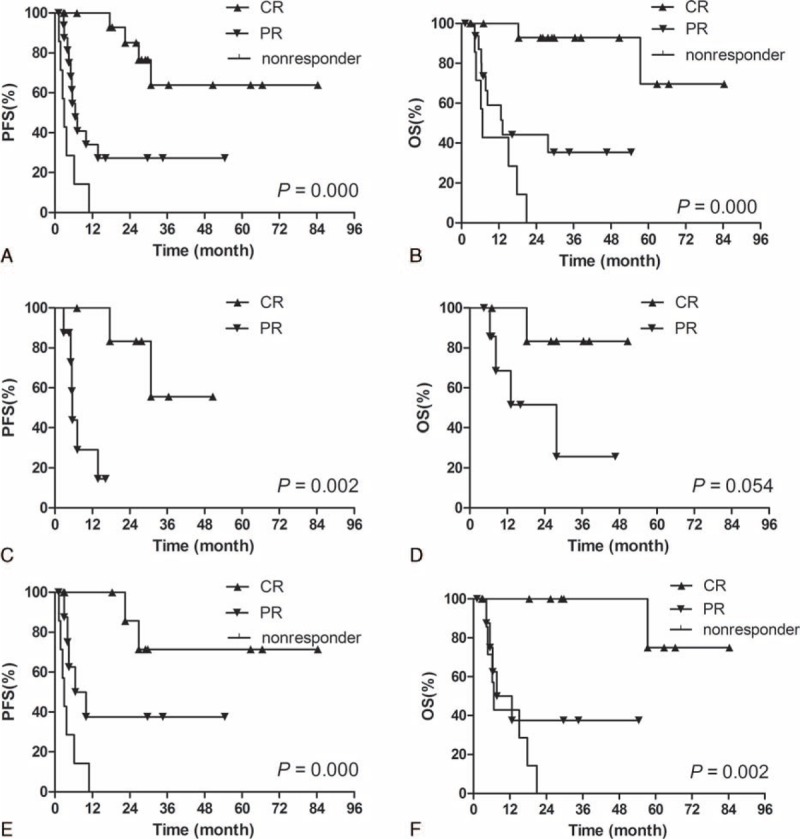
Kaplan–Meier analysis of PFS (A) and OS (B) for all patients according to chemotherapy response; Kaplan–Meier analysis of PFS (C) and OS (D) for newly diagnosed stage IV patients according to chemotherapy response; Kaplan–Meier analysis of PFS (E) and OS (F) for relapsed/refractory patients according to chemotherapy response. OS = overall survival, PFS = progression-free survival.

CR, PR, and no response (SD plus PD) were significant prognostic factors against PFS and OS: 1-year PFS: 92.9% versus 34.1% versus 0.0% and 2-year PFS: 85.1% versus 27.3% versus 0.0%, *P* = 0.000; 1-year OS: 92.9% versus 58.9% versus 42.9% and 2-year OS: 92.9% versus 44.2% versus 0.0%, *P* = 0.000 (Figure 2A and B). Chemotherapy responses were also significant factors for PFS and OS in the subgroup of newly diagnosed stage IV patients and the subgroup of relapsed/refractory patients, respectively (Figure 2C–F). Univariate analysis revealed ALC before chemotherapy to be significant factors for PFS and OS of the whole cohorts (Table 2).

### Toxicity

Treatment-related toxicities were summarized in Table 3. The most common grade 3 to 4 toxicity was neutropenia, which was observed in 14 (34.1%) patients. Grade 3 to 4 thrombocytopenia was observed in 8 (19.5%) patients. Grade 1 to 2 nausea and vomiting were the most common nonhematological toxicities, observed in 87.8% of patients. No grade 3 to 4 nonhematological toxicities were observed. There were no treatment-related deaths. Six (14.6%) patients experienced 25% dose reduction because of serious hematological toxicities and the median number of experiencing reduction was the fourth cycle.

**TABLE 3:**
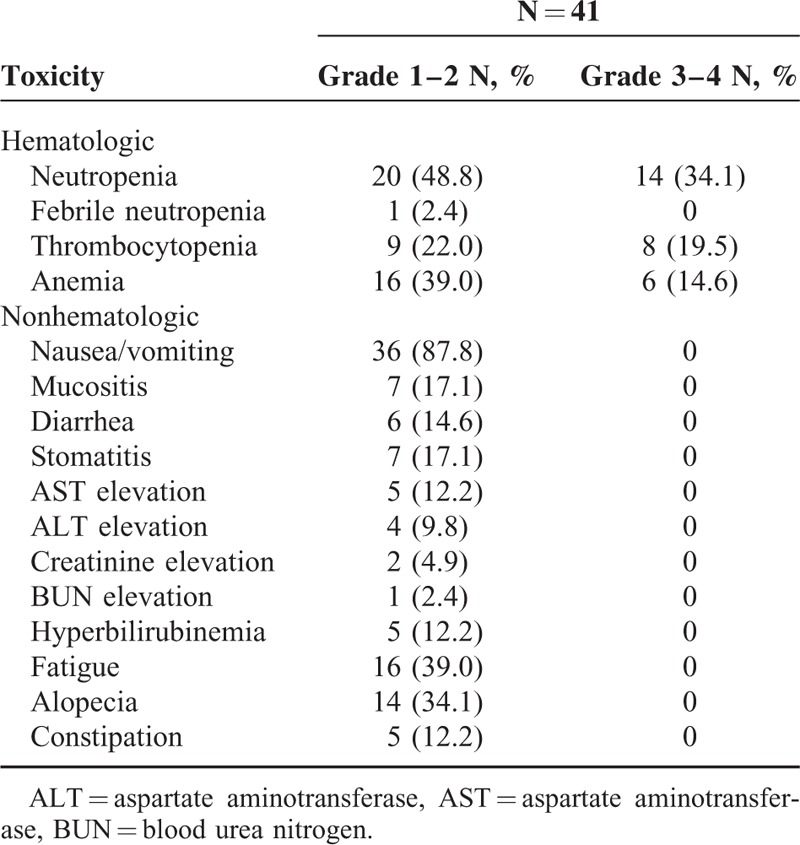
Toxicity Profile

## DISCUSSION

Our study demonstrates that GDP chemotherapy is effective and well-tolerated in the treatment of newly diagnosed stage IV and relapsed/refractory ENKTL. The ORR was 82.9% with 41.5% CR rate. The 1- and 2-year PFS rates were 54.5% and 45.4%; the 1- and 2-year OS rates were 72.7% and 54.7%. Grade 3 to 4 adverse events included neutropenia (34.1%), thrombocytopenia (19.5%), and anemia (14.6%).

Gemcitabine is an analog of cytosine arabinoside, but is more effectively taken up into cells, phosphorylated, and retained intracellularly.^17^ A series of researches reported the efficacy of gemcitabine-based chemotherapy regimens. In a prospective multicenter study,^18^ 619 patients with relapsed/refractory aggressive lymphoma were randomly assigned to treatment with GDP or to dexamethasone, cytarabine, and cisplatin. Patients with B-cell lymphoma also received rituximab. The ORRs after 2 treatment cycles were 45.2% and 44.0%, respectively. Similar event-free survival and OS were observed, and GDP was associated with less toxicity and preserved quality of life. In another prospective clinical trial,^19^ 44 patients with relapsed/refractory Hodgkin lymphoma were randomly divided into 2 groups that were treated with GDP and etoposide, methyl prednisolone, cisplatin, and cytarabine. Similar response rate were obtained (59% vs 55%). In a prospective phase II study, Park et al^20^ reported GDP was a highly effective and optional salvage regimen for relapsed/refractory PTCLs. The ORR was 72% with 48% CR rate. In addition, Zhong et al^21^ reported GDP regimen yielded an ORR of 54% in relapsed/refractory AIDS-related NHL and did not promote HIV-1 viral replication.

As for the treatment of ENKTL, Ahn et al^22^ reported that using gemcitabine alone or containing chemotherapy for relapsed/refractory disease, 8 of 20 patients (40%) achieved response. Given that 5 of 8 responders had received L-asparaginase-containing chemotherapy in preceding treatment, this response rate was quite promising. In our previous study,^12^ an ORR of 86% was observed for ENKTL patients with GDP and dexamethasone, ifosfamide, methotrexate and gemcitabine combination regimens. In addition, the Zhang group adopted the gemcitabine, pegaspargase, cisplatin, and dexamethasone regimen to treat 17 relapsed/refractory ENKTL patients^23^ and 12 newly diagnosed ENKTL patients;^24^ the ORRs were 88% and 100%, respectively. Therefore, gemcitabine-based regimens might be a promising option for the treatment of ENKTL patients.

Patients with advanced-stage and relapsed/refractory disease have a poor prognosis,^25–28^ and no standard management is identified for relapsed/refractory patients. In some studies, L-asparaginase-based treatments yielded high ORR of 79%–90% in disseminated and relapsed/refractory patients and consequent survival benefit was observed Table 4.^29–32^ In our study, the ORR (83.0%) and CR rate (41.5%) of all patients were comparable to the respective rates reported for L-asparaginase-containing combination regimens. Higher ORR was achieved in newly diagnosed patients than in relapsed/refractory patients (100% vs 73%, *P* = 0.035), consistent with the results of gemcitabine, pegaspargase, cisplatin, and dexamethasone regimen.^23,24^ Therefore, our results suggest that GDP regimen is more appropriate for newly diagnosed ENKTL patients.

**TABLE 4:**
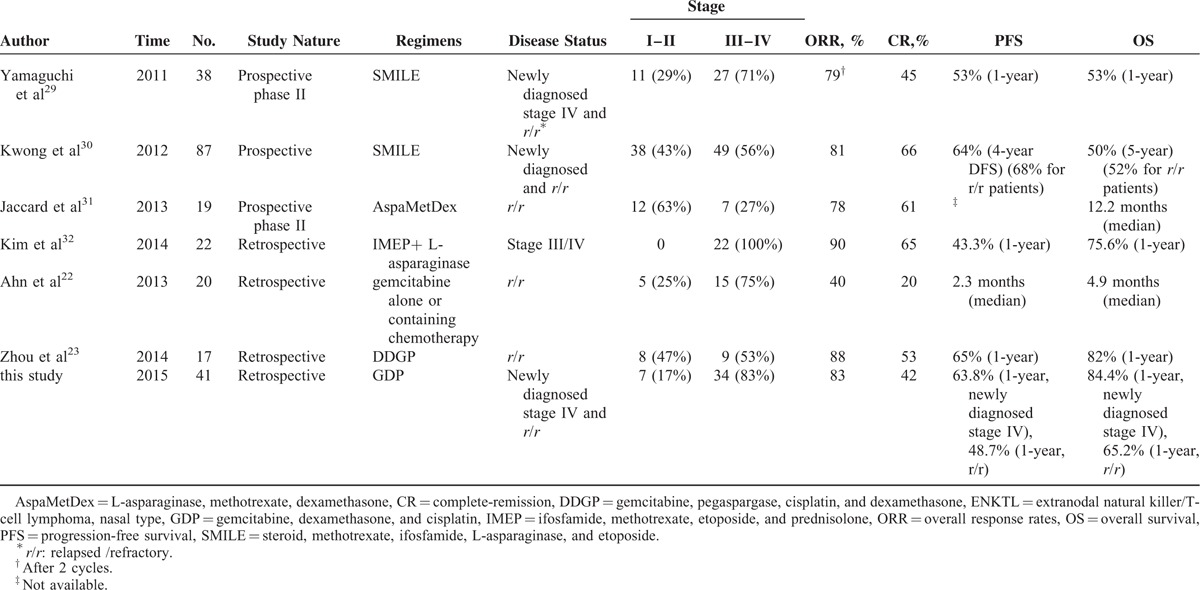
L-Asparaginase-Based Regimens and Gemcitabine-Based Regimens for Advanced-Stage and Relapsed/Refractory ENKTL Patients

In our study, comparable PFS (1-year: 55% and 2-year: 45%) and OS (1-year: 73% and 2-year: 55%) to L-asparaginase-containing regimens were achieved for advanced-stage or relapsed/refractory ENKTL patients, Table 4^29–32^ although long-term follow-up is necessary. In the study of Kim et al,^32^ PFS and OS were significantly higher for the IMEP (ifosfamide, methotrexate, etoposide, and prednisolone) plus L-asparaginase group compared with the IMEP group for the treatment of advanced-stage ENKTL patients (1-year PFS: 43% vs 17%, *P* = 0.002; 1-year OS: 76% vs 38%, *P* = 0.006). In another retrospective study, better PFS was found in the modified SMILE regimen group compared with the CHOP group in advanced-stage or relapsed/refractory patients.^33^ In these 2 studies, the worse survival rates of control groups may be partly attributed to the low efficacy of IMEP regimen (ORR: 35%) and CHOP regimen (ORR: 36%), whereas GDP regimen yielded high ORR of 83% in our study. Therefore, more studies are needed to identify whether the combination of L-asparaginase and GDP can further improve survival of patients with newly diagnosed and relapsed/refractory ENKTL.

Generally, GDP regimen was well-tolerated, and the primary toxicity was myelosuppression. We postulate 2 reasons for the low-toxicity profile of the treatment regimen, including the lack of overlapping toxicities among gemcitabine, cisplatin, and dexamethasone, as well as the timing of dexamethasone administration. Dexamethasone used on days 1 to 4 and days 11 to 14 caused a marked increase in circulating polymorphonuclear leukocytes, so gemcitabine of day 8 could be administered on schedule. Kwong et al^30^ reported 87 ENKTL patients treated with SMILE regimen. Serious infections occurred in 27 (31%) patients, with 8 (9%) patients requiring admission into intensive care unit, leading to 5 (6%) deaths. In the phase II study of SMILE regimen,^29^ 2 (5%) of the 38 patients died from grade 5 infections, and grade 3 to 4 leukopenia was observed in all 38 (100%) patients. In our study, there were no treatment-related deaths; grade 3 to 4 neutropenia was observed in 14 (34%) patients. In addition, compared with L-asparaginase-containing combination regimens, GDP regimen has lower incidence of prolonged activated partial thromboplastin time, pancreatitis, and anaphylactic reaction. These results indicate that the tolerability of GDP is better than L-asparaginase-containing regimens while maintaining similar therapeutic effects.

This study is limited by its retrospective nature, nonrandomized design, and small sample size. Given the rarity of this disease and consequent lack of prospective data, the present study provides evidence to confirm GDP regimen as highly effective treatment in ENKTL patients. Further prospective studies are needed to validate the results. Considering that ENKTL is mostly diagnosed at early stage, further randomized controlled trials are warranted to identify whether the integration of GDP with appropriate radiotherapy could reduce systemic failure and improve survival rates of early-stage patients.

In conclusion, GDP is a highly effective regimen for ENKTL patients with markedly less toxicities. It may be a treatment of choice for newly diagnosed stage IV and relapsed/refractory ENKTL patients.
